# Prediction of suicidal ideation among Chinese college students based on radial basis function neural network

**DOI:** 10.3389/fpubh.2022.1042218

**Published:** 2022-12-01

**Authors:** Shiyi Liao, Yang Wang, Xiaonan Zhou, Qin Zhao, Xiaojing Li, Wanjun Guo, Xiaoyi Ji, Qiuyue Lv, Yunyang Zhang, Yamin Zhang, Wei Deng, Ting Chen, Tao Li, Peiyuan Qiu

**Affiliations:** ^1^Department of Epidemiology and Statistics, West China School of Public Health, Sichuan University, Chengdu, Sichuan, China; ^2^Ministry of Education Key Laboratory of Child Development and Disorders, Chongqing Key Laboratory of Pediatrics, National Clinical Research Center for Child Health and Disorders, Children's Hospital of Chongqing Medical University, Chongqing, China; ^3^Department of Neurobiology and Affiliated Mental Health Center & Hangzhou Seventh People's Hospital, Zhejiang University School of Medicine, Hangzhou, Zhejiang, China; ^4^Mental Health Center and Psychiatric Laboratory, West China Hospital of Sichuan University, Chengdu, Sichuan, China; ^5^West China School of Public Health, Sichuan University, Chengdu, Sichuan, China

**Keywords:** suicidal ideation, radial basis function neural network (RBFNN), prediction, college student, China

## Abstract

**Background:**

Suicide is one of the leading causes of death for college students. The predictors of suicidal ideation among college students are inconsistent and few studies have systematically investigated psychological symptoms of college students to predict suicide. Therefore, this study aims to develop a suicidal ideation prediction model and explore important predictors of suicidal ideation among college students in China.

**Methods:**

We recruited 1,500 college students of Sichuan University and followed up for 4 years. Demographic information, behavioral and psychological information of the participants were collected using computer-based questionnaires. The Radial Basis Function Neural Network (RBFNN) method was used to develop three suicidal ideation risk prediction models and to identify important predictive factors for suicidal ideation among college students.

**Results:**

The incidence of suicidal ideation among college students in the last 12 months ranged from 3.00 to 4.07%. The prediction accuracies of all the three models were over 91.7%. The area under curve scores were up to 0.96. Previous suicidal ideation and poor subjective sleep quality were the most robust predictors. Poor self-rated mental health has also been identified to be an important predictor. Paranoid symptom, internet addiction, poor self-rated physical health, poor self-rated overall health, emotional abuse, low average annual household income per person and heavy study pressure were potential predictors for suicidal ideation.

**Conclusions:**

The study suggested that the RBFNN method was accurate in predicting suicidal ideation. And students who have ever had previous suicidal ideation and poor sleep quality should be paid consistent attention to.

## Introduction

Suicide is an important public health issue worldwide. The World Health Organization (WHO) estimated that about 703,000 people died by suicide every year (1). Among people age 15–29 year old, suicide is the fourth leading cause of death (2). And suicide has become the leading cause of death in the Chinese population ages 15–34 (3), an age group that covers college students. The prevalence of suicide death among college students was about 4.7 per 100,000 students (4). Suicidal behaviors include suicide ideation, suicide planning, suicide attempting and suicide (5). Suicide ideation is defined as thoughts about engaging in suicidal behavior (6). The pooled prevalence estimates of suicidal ideation, plans, and attempts worldwide were 10.6, 3.0, and 1.2%, respectively (7). In China, the pooled prevalence estimates of suicidal ideation, plans, and attempts were 10.72% (8), 4.4 % (9), and 2.8% (3), respectively.

As the first step toward suicide, suicidal ideation has been identified as an important precursor to suicide (10). A Meta-Analysis included 51 studies reported that individuals with suicidal ideation were over three times more likely to commit suicide than those without suicidal ideation (11). Therefore, the investigation of suicidal ideation and its predictors may help identify college students at high risk for suicide and enable early intervention to prevent suicide. Previous studies indicated that past lifetime suicidal ideation and attempt, poor self-reported physical and mental health, sleep disturbances, loneliness, stressful life events, childhood/adolescence abuse and neglect were potential risk factors of suicidal ideation in general population (12–15). Compared with other periods, college students experience a critical transition period, which is characterized by the stress from adapting to a new environment, losing and rebuilding social support systems. Therefore, besides aforementioned risk factors, study pressure, having been bullied, academic difficulties, and substance use were suggested by previous studies as risk factors for suicidal ideation among college students (16–20). However, the results of which predictors are most useful were inconsistent due to different sample populations, statistical methods and questionnaires.

Traditionally, structural equation modeling, Pearson's correlation analysis and conventional linear models were often used to identify risk factors for suicidal ideation. However, a series of studies found that machine learning was doing a better job than traditional methods to predict suicidal ideation (21–23). A meta-analysis including 365 studies found that existing traditional methods worked only slightly better than chance to predict suicidal thoughts and behaviors (23). While machine learning could predict suicidal behavior with 40%−60% better prediction than chance (24–27). Machine learning has been increasingly showing advantages over traditional statistical methods in terms of accuracy and scalability (21). First, machine learning methods can map a target outcome to factors of interest with the most accurate and parsimonious algorithm (21, 28). Except parameters being adjusted by the researchers, the optimal path through the data is mostly determined by the machine. By contrast, traditional approaches require a pre-programmed algorithms which largely rely on prior hypotheses proposed by researchers (21, 28, 29). Therefore, the algorithms of traditional approaches were considered to be quite simple, which usually used a small set of predictors combined in a fairly basic way. Second, given advances in computing power, machine learning algorithms allow for the simultaneous testing of numerous factors and their complex interactions (21). Yet traditional approaches fail to accommodate a large number of factors or make complex combinations due to the reasons discussed above. Third, when dealing with high-dimensional datasets that include a large number of potential predictors, machine learning algorithms have shown better performances in preventing overfitting, comparing to traditional statistical approaches to such data is easily to overfitting (21). The artificial neural network (ANN) is a branch of artificial intelligence duplicating the biological brain systems (30). One of the main advantages of ANN is self-learning without prior knowledge of the complex relationships that exist between the input and output variables (31). Other advantage is that ANN is used effectively to approximate non-linear functions and can be trained for multi-dimensional variables (32). As one of neural network learning methods, radial basis function neural network (RBFNN) is an efficient single-hidden layer forward network, which mimics the neural network structure of local regulation and mutual coverage of sensory domains in the human brain (33). RBFNN has been proven to possess the universal approximation ability and no local minimum problem (34, 35). Moreover, it has a simpler structure, a deeper physiological foundation and faster learning ability compared to other neural networks (36). Nowadays, RBFNN has been widely used in forecasts, such as regional GDP forecasting, stock market forecasting and predicting the level of disinfection by-products in tap water (37–39).

Although there have been studies on predictors of suicidal ideation, the results have been inconsistent and few studies have systematically investigated psychological symptom of participants, which are important predictors of suicidal ideation. Along with the limitations of traditional methods in predicting suicide ideation, this study aims to employ RBFNN to develop a suicidal ideation prediction model and explore important predictors of suicidal ideation among college students in China.

## Materials and methods

### Sample

The University Students Study of Sichuan Province is a longitudinal investigation into psychological symptoms of university students through 2014 to 2018. It was carried out in the Sichuan University, a comprehensive university in the southwest China. We recruited all the freshmen who entered the school in 2014 and 2015. To make the best use of the database while maintaining sufficient observation points, we combine new recruitments from 2014 and 2015 waves as our baseline sample. For participants who were recruited from 2014, follow-up investigation in 2015, 2016, and 2017 were treated as wave 2, wave 3, and wave 4, respectively. For participants who were recruited from 2015, follow-up investigation in 2016, 2017, and 2018 were treated as wave 2, wave 3 and wave 4, respectively. Each student has a unique login ID, and complete the questionnaires on the computer on their own. The online questionnaires were distributed in the 1–3 months of the school year. The inclusive criteria for this study were completion of investigation of all four waves and approval of participation. We recruited 17,405 participants at baseline, and excluded 15,905 participants due to unmet inclusive criteria and important variables missing. The final sample size was 1,500. The detailed information of follow-up among participants was described in the Supplementary Figure S1.

### Measures

In our study, suicide ideation in the last 12 months was the outcome variable. It was measured with one question: Have you ever thought about killing yourself in the last 12 months? Participants who answered “Yes” were coded as having suicide ideation in the last 12 months.

Predictors collected in this study included: (1) demographic information, including gender, age, income (average annual household income per person); (2) previous suicidal ideation; (3) self-rated overall health; (4) physical health information, including self-rated physical health, chronic disease, number of medical visits in the previous 12 months and somatic symptoms; (5) mental health, including family history of mental or psychological illness, self-rated mental health, hypochondriasis, psychological distress, paranoid symptom, psychotic symptom, depressive symptoms, subjective sleep quality, sleep disturbance, compulsion, and internet addiction; (6) negative life events, including interpersonal relationships, study pressure, punishment, sense of loss, change for adaptation, other stressful life events, physical abuse, emotional abuse, sexual abuse, physical neglect and emotional neglect. Assessment tools are reported as follows.

#### PHQ-15 scale

The Patient Health Questionaire-15 (PHQ-15) is a continuous measure of somatic symptoms in the past month. It contains 15 items rated on a 3-point Likert scale (0: not bothered at all−2: bothered a lot). The total score ranges from 0 to 30. A higher score refers to severer somatic symptoms (40).

#### Hypochondriasis scale

The Hypochondriasis Scale is a self-designed tool to evaluate an individual's predisposition to hypochondriasis over the past month. The scale has seven items rated on a 5-point Likert scale (0: no distress at all−4: heavy distress). Total score of the scale is between 0 and 28.

#### K-6 scale

Kessler Psychological Distress Scale (K-6) is used to examine psychological distress over the last 30 days (41). K-6 has six items in total, four of which measure depressive symptoms and the other two items test anxiety symptoms. Answers are scored on a 5-point Likert scale (0: none of the time−4: all of the time). The total score ranges from 0 to 24 (42).

#### ASLEC scale

The frequency of stressful life events and stress response intensity was measured by the Adolescents Self-Rating Life Events Checklist (ASLEC) (43). The ASLEC consists of 27 items of negative life events, including six dimensions: interpersonal relationships, study pressure, punishment, sense of loss, change for adaptation, and others. When there were no negative life events, the score is 0 (not occur). If negative life events happened, a 5-point Likert scale (1: no impact at all to 5: very strong impact) is needed to be answered. A higher score indicates greater stress (44).

#### CTQ

Childhood Trauma Questionnaire (CTQ) was used to assess participants' exposure to neglect and abuse during childhood. The CTQ consists 28 items, including 25 clinical items and three validity items. The 25 clinical items can be divided in five dimensions: physical abuse, emotional abuse, sexual abuse, physical neglect and emotional neglect. The items are rated on a 5-point Likert scale (0: never−4: always). Each dimension consists of five items, with a total score between 0 and 20. Higher total score indicates more severe childhood abuse or neglect (45).

#### SCL-90-R

The Symptom Checklist-90-Revised (SCL-90-R) is a useful tool to evaluate psychotic experiences. Two symptom dimensions relevant to psychosis include six items in the paranoid ideation and 10 items in the psychoticism. Each item is rated on a 5-point Likert scale (0: not at all−4: extremely) (46). The total score ranges from 0 to 24 in paranoia subscales. The higher the total score, the more severe the paranoid symptom. The total score of psychoticism is between 0 and 40, with higher total scores indicating more severe psychotic symptoms.

#### PHQ-9

Patient Health Questionnaire-9 (PHQ-9) explores the depressive symptoms experienced by patients over the past 2 weeks. The PHQ-9 consists of nine questions rated on 4-point Likert scale (0: not at all−3: nearly every day). The total sum score ranges from 0 to 27, with higher scores indicating higher levels of depressive symptoms (47).

#### PSQI

The Pittsburgh Sleep Quality Index (PSQI) assesses sleep quality over a 1-month period. The PSQI scale is categorized into seven dimensions. We only investigated two dimensions of it, including subjective sleep quality and sleep disturbance. Subjective sleep quality has one item, using a 4-point Likert scale (0: very good−3: very poor). Sleep disturbance includes 12 items rated on a 5-point Likert scale (0: none−4: almost every day). The total score of sleep disturbance ranges from 0 to 48. Higher scores indicate worse sleep quality (48).

#### OCI-R

The Obsessive-Compulsive Inventory-revised (OCI-R) is used to assess the distress associated with obsessions and compulsions. OCI-R consists of 18 items, including six dimensions: washing, checking, ordering, obsessing, hoarding and neutralizing symptom clusters. Items are rated on a 5-point Likert scale (0: none−4: extremely frequent). The total score ranges from 0 to 72, and each dimension score ranges from 0 to 12. Higher scores represent higher levels of Obsessive–compulsive symptoms (49).

#### IAT

The Internet Addiction Test (IAT) was developed by Kimberly Young to assess psychological dependence, compulsive use, and withdrawal symptoms resulting from excessive internet use. The IAT consists of 20 questions on 5-point Likert scale (1: rarely−5: always), with a sum of scores from 20 to 100. Higher scores represent a severer state of internet addiction (50).

#### Self-rated health

Three questions were used to assess self-rated health, including self-rated physical health, self-rated mental health and self-rated overall health. Each item is graded on a 5-point Likert scale (0: perfect−4: poor).

### Statistical analysis

In this study, three prediction models were established to predict the suicidal ideation of college students in the next year respectively. Model 1 used the predictors in wave one to predict suicidal ideation in wave two. Model 2 used the variables in wave two to predict the suicidal ideation in wave three. Model 3 used the variables in wave three to predict the suicidal ideation in wave four. Predictors for each model were shown in Table 1.

**Table 1:** Predictors of three models.

**Predictors**	**Model 1**	**Model 2**	**Model 3**
Gender	√	√	√
Age	√	√	√
Income (average annual household income per person)	√	√	√
Family history of mental or psychological illness	√	√	√
Chronic physical illness	√	√	√
Number of medical visits in last year	√	√	√
Previous suicidal ideation	√	√	√
PHQ-15	√	√	√
Hypochondriasis Scale	√	–^*^	–^*^
K-6	√	√	√
**ASLEC**
Interpersonal relationships	√	√	√
Study pressure	√	√	√
Punishment	√	√	√
Sense of loss	√	√	√
Change for adaptation	√	√	√
Others	√	√	√
**CTQ**
Physical abuse	√	√	√
Emotional abuse	√	√	√
Sexual abuse	√	√	√
Physical neglect	√	√	√
Emotional neglect	√	√	√
**SCL-90-R**
Paranoid symptom	√	√	√
Psychotic symptom	√	√	√
Previous suicidal ideation	√	√	√
PHQ-9	√	√	√
**PSQI**
Subjective sleep quality	√	√	√
Sleep disturbances	√	√	√
OCI-R	–^*^	√	√
IAT	√	√	√
**Self-rated health status**
Physical health	–^*^	√	√
Mental health	–^*^	√	√
Overall health	–^*^	√	√
Numbers of predictors	27	30	30

* Scales were not used in the corresponding wave.

We applied RBFNN to establish a suicidal ideation risk prediction model using python 3.6.8. RBFNN is a three-layer artificial neural network, including input layer, hidden layer and output layer (51). The number of nodes in the input layer is equal to the dimensions of the input variables. The hidden layer's number of nodes is determined according to the complexity of the problem. The number of nodes in output layer is equal to the dimensions of the output variables. In this study, the input layer is a matrix composed of predictors of suicidal ideation among college students, while the output layer has only one output variable that is the risk of suicidal ideation in a year among college students.

We first pre-processed data and performed feature selection. In this study, literature review method and expert consultation method were adopted to screen relevant variables as predictors of RBFNN modeling. Second, we randomly selected 300 participants as the testing set. The remaining dataset was randomly split into a 75% training set and a 25% validation set. The training set was used for training a neural network and the validation set was employed to verify the network's performance over training. The testing set was used to assess the accuracy and predictability of the model (52). Ten-fold cross-validation was used to assess predictive performance and general error estimates in the machine learning process. Third, the gradient descent method was used to select center parameters of hidden layer neurons, and the number of iterations in this study was determined by an early stopping method. The parameters of the prediction model of RBFNN were finally determined as follows: the number of hidden layer nodes was 60, the learning rate was 0.08, and the number of iterations was 100. Under this parameter setting, the average prediction accuracy of the four validation sets reached the highest. Fourth, a series of indicators, including classification accuracy, sensitivity, specificity, positive predictive value (PPV), negative predictive value (NPV), G-mean value and Area under ROC Curve (AUC) were used to evaluate the prediction effect of the models. Accuracy is the percentage of the number of people with correct classification in the total number of people in the prediction model. Sensitivity reflects the model's ability to correctly identify the positive incidents, while specificity refers to the percentage of the correctly predicted negative incidents. PPV measures the ratio of true positive predictions considering all positive predictions. NPV measures the ratio of true negative predictions considering all negative predictions. G-mean is often used to evaluate the effect of prediction classification model in unbalanced data (53). An AUC of 1.0 represents a perfect test, with no false positive rate and no false negative rate, while an AUC of 0.5 indicates that the test performed no better than chance (54). Moreover, we used mean impact value (MIV) to identify important predictors for suicidal ideation (55).

## Results

### Sample characteristics

A total of 1,500 participants were included. At wave one, the average age was 18.22 ± 0.76 years old. Among the 1,500 participants, 43.67% were men, 97.73% had no family history of mental illness, 89.73% had no chronic physical diseases, 50.40% had an average annual household income per person <5,000 yuan. The average number of medical visits in last year was 2.05 ± 2.80. Through the four waves, the incidences of suicidal ideation in the last 12 months were 3.33, 3.00, 3.00, and 4.07%, respectively. The detailed information was described in Table 2. We compared the incidence of suicidal ideation between the sample population and those who were lost to follow-up and there was no significant difference. The detailed information was described in the Supplementary Table S1.

**Table 2:** Distribution of main variables through wave one to wave four.

**Variable**	**Different stages of investigation** ***N*** **(%) or** ***M*****(*****SD*****)**			
	**Wave 1**	**Wave 2**	**Wave 3**	**Wave 4**
**Suicidal ideation occurred in the last 12 months**	50 (3.33)	45 (3.00)	45 (3.00)	61 (4.07)
**Gender**
Male	655 (43.67)	655 (43.67)	655 (43.67)	655 (43.67)
Female	845 (56.33)	845 (56.33)	845 (56.33)	845 (56.33)
**Income (average annual household income per person)**
Less than 3,000 yuan	369 (24.60)	369 (24.60)	369 (24.60)	369 (24.60)
Between 3,000~5,000 yuan	387 (25.80)	387 (25.80)	387 (25.80)	387 (25.80)
Between 5,000~10,000 yuan	273 (18.20)	273 (18.20)	273 (18.20)	273 (18.20)
Between 10,000~20,000 yuan	182 (12.13)	182 (12.13)	182 (12.13)	182 (12.13)
Between 20,000~30,000 yuan	158 (10.53)	158 (10.53)	158 (10.53)	158 (10.53)
Beyond 30,000 yuan	131 (8.73)	131 (8.73)	131 (8.73)	131 (8.73)
**Family history of mental or psychological illness**
Yes	34 (2.27)	34 (2.27)	34 (2.27)	34 (2.27)
No	1,466 (97.73)	1,466 (97.73)	1,466 (97.73)	1,466 (97.73)
**Chronic physical diseases**
0	1,346 (89.73)	1,229 (81.93)	1,193 (79.53)	1,189 (79.27)
1	122 (8.13)	227 (15.13)	250 (16.67)	256 (17.07)
2	27 (1.80)	34 (2.27)	46 (3.07)	46 (3.07)
≥3	5 (0.33)	10 (0.67)	11 (0.73)	9 (0.60)
**Number of medical visits in last year**	2.05 (2.80)	1.48 (2.48)	1.60 (2.38)	1.61 (2.49)
**Previous suicidal ideation**	236 (15.73)	256 (17.07)	271 (18.07)	294 (19.60)
**PHQ-15**	2.12 (2.18)	2.68 (2.60)	2.74 (2.76)	2.39 (2.52)
**Hypochondriac scale**	1.39 (2.25)	–	–	–
**K6**	2.95 (2.79)	3.43 (3.43)	3.42 (3.38)	3.61 (3.91)
**ASLEC**
Interpersonal relationships	3.22 (3.45)	2.66 (3.25)	2.41 (3.13)	1.98 (2.90)
Study pressure	4.44 (3.39)	3.86 (3.41)	4.46 (3.78)	4.14 (3.82)
Punishment	1.66 (2.94)	1.43 (2.65)	1.33 (2.40)	0.99 (2.02)
Sense of loss	1.13 (2.05)	0.93 (1.77)	0.84 (1.71)	0.69 (1.51)
Change for adaptation	2.39 (1.83)	1.83 (1.89)	1.68 (1.81)	1.07 (1.85)
Others	1.05 (1.81)	1.28 (1.96)	1.21 (1.78)	0.92 (1.66)
Total	13.54 (11.43)	11.70 (11.62)	11.64 (11.28)	9.57 (10.44)
**CTQ**
Physical abuse	0.28 (0.96)	0.28 (0.96)	0.28 (0.96)	0.28 (0.96)
Emotional abuse	0.81 (1.56)	0.81 (1.56)	0.81 (1.56)	0.81 (1.56)
Sexual abuse	0.17 (0.76)	0.17 (0.76)	0.17 (0.76)	0.17 (0.76)
Physical neglect	2.87 (3.00)	2.87 (3.00)	2.87 (3.00)	2.87 (3.00)
Emotional neglect	5.96 (6.63)	5.96 (6.63)	5.96 (6.63)	5.96 (6.63)
Total	10.10 (9.78)	10.10 (9.78)	10.10 (9.78)	10.10 (9.78)
**SCL-90-R**
Paranoid symptom	1.64 (2.30)	1.41 (2.19)	1.24 (2.11)	0.93 (1.90)
Psychotic symptom	2.32 (3.25)	2.24 (3.46)	2.03 (3.42)	1.48 (2.92)
**PHQ-9**	3.05 (3.18)	3.33 (3.53)	3.30 (3.64)	2.95 (3.69)
**PSQI**
Subjective sleep quality	0.88 (0.69)	0.96 (0.73)	0.93 (0.70)	0.92 (0.71)
Sleep disturbance	3.32 (3.74)	3.67 (4.13)	3.92 (4.40)	3.57 (4.32)
**OCI-R**	3.23 (2.58)	5.59 (6.97)	5.35 (6.88)	4.46 (6.21)
**IAT**	11.30 (9.79)	12.77 (11.84)	11.80 (11.15)	10.51 (11.78)
**Self-rated health status**
Physical health	–^*^	1.43 (0.91)	1.42 (0.93)	1.38 (0.94)
Mental health	–^*^	1.26 (0.92)	1.25 (0.95)	1.26 (0.96)
Overall health	–^*^	1.29 (0.87)	1.30 (0.90)	1.26 (0.90)

* Scales were not used in the corresponding wave.

### Prediction of suicidal ideation among college students in the following year

**Figure 1:**
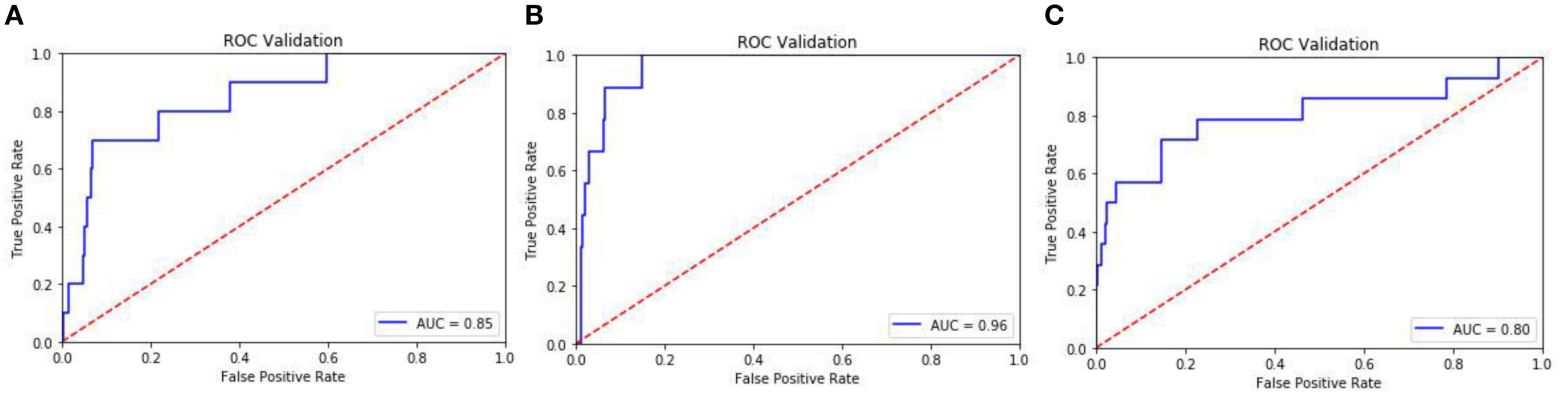
The ROC curves of the testing set. **(A)** ROC curve of the testing set in model 1. **(B)** ROC curve of the testing set in model 2. **(C)** ROC curve of the testing set in model 3.

The accuracy, sensitivity, specificity, PPV, NPV, G-mean and AUC of the three models were shown in Table 3. The accuracy ranged from 0.917 to 0.953, showing that <10% of participants were misclassified by the models using the selected set of variables. The sensitivity ranged from 0.500 to 0.677, suggesting that more than 50% of participants who actually had suicidal ideation were predicted to be those who had suicidal ideation. The specificity ranged from 0.934 to 0.962, indicating that about 94.4% of participants who actually did not have suicidal ideation were correctly predicted. PPV ranged from 0.208 to 0.353, suggesting that 20.8%−35.3% of the participants classified as having suicidal ideation by model were actually those who reported a suicidal ideation. NPV ranged from 0.978 to 0.989, indicating that more than 97.8% of participants considered as not having suicidal ideation by model were actually those who didn't report a suicidal ideation. G-mean of three models were 0.684, 0.801, and 0.730, respectively. The AUC ranged from 0.80 to 0.96, reflecting a moderately good discrimination (Figure 1).

**Table 3:** Discrimination performances for the prediction models.

**Evaluation index (95%CI)**	**Model 1**	**Model 2**	**Model 3**
Accuracy	0.920 (0.883–0.946)	0.953 (0.923–0.973)	0.917 (0.879–0.943)
Sensitivity	0.500 (0.201–0.799)	0.667 (0.309–0.910)	0.571 (0.296–0.812)
Specificity	0.935 (0.898–0.959)	0.962 (0.931–0.980)	0.934 (0.897–0.958)
PPV	0.208 (0.079–0.427)	0.353 (0.153–0.614)	0.296 (0.145–0.503)
NPV	0.982 (0.956–0.993)	0.989 (0.967–0.997)	0.978 (0.950–0.991)
AUC	0.85 (0.70–1.0)	0.96 (0.87–1.0)	0.80 (0.66–0.94)

**Figure 2:**
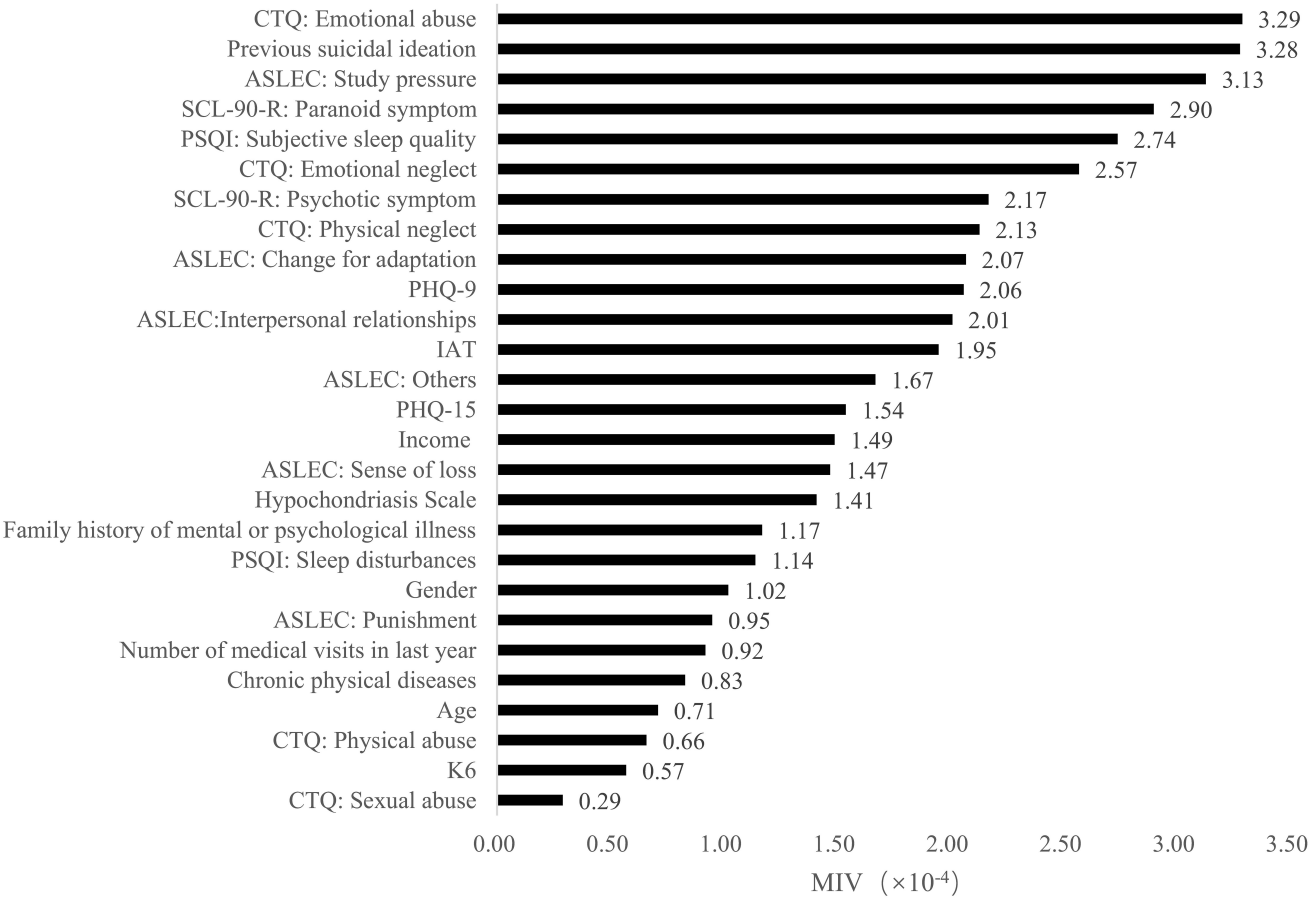
The mean impact values of independent variables in model 1.

**Figure 3:**
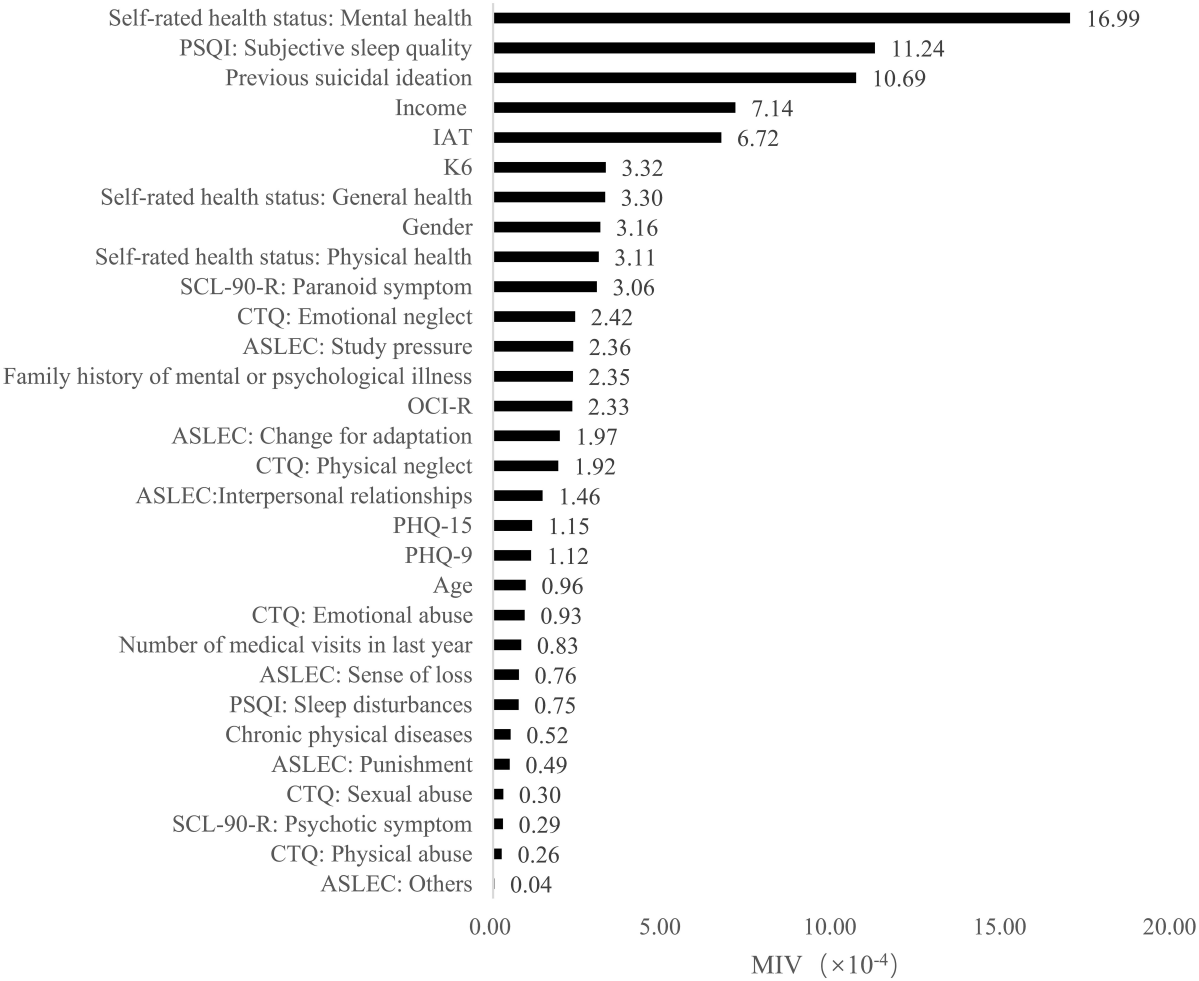
The mean impact values of independent variables in model 2.

**Figure 4:**
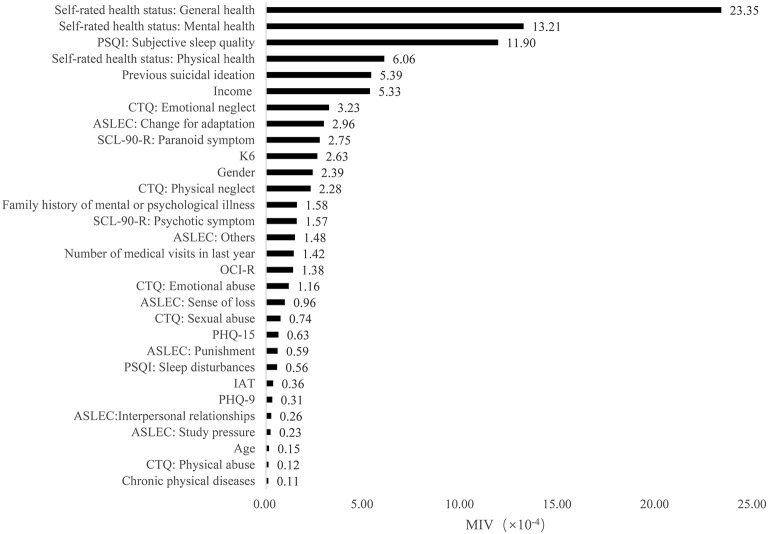
The mean impact values of independent variables in model 3.

Furthermore, based on the results of MIVs, we sorted the predictors in each model from the most important to the least important. In model 1, the top five predictors were emotional abuse, previous suicidal ideation, study pressure, paranoid symptom and poor subjective sleep quality. In model 2, the most important five predictors were self-rated mental health, poor subjective sleep quality, previous suicidal ideation, income and internet addiction. In model 3, self-rated overall health, self-rated mental health, poor subjective sleep quality, self-rated physical health and previous suicidal ideation were the top five predictors. The MIVs of independent variables were present in Figures 2–4.

## Discussion

We found that the prevalence of suicidal ideation ranged from 3.00 to 4.07% among college students of Sichuan University in the last 12 months, which is similar to the prevalence found by Wang (4.21% among freshmen in college in Henan) (56), and Chen (5.3% among undergraduate students in Jilin) (57) in China. The reported prevalence rates of suicidal ideation in many other countries were between 9.7 to 58.3% (58), which were higher than that in our study. This might be related to different cultural contexts and different survey scales.

In our study, we developed three RBFNN prediction models to predict the suicidal ideation in the next year among college students. All three models had high prediction accuracy (from 0.917 to 0.953), moderate sensitivity (from 0.500 to 0.667), high specificity (from 0.934 to 0.962), moderate G-mean (from 0.648 to 0.801) and high AUC (from 0.80 to 0.96). The RBFNN prediction model reflected a moderately good discrimination (e.g. AUCs in the 0.8s−0.9s range) (59).

We found in our study that previous suicidal ideation and poor sleep quality were the most important predictors for suicidal ideation in the last 12 months in all the three models. The results were consistent with previous studies. Catharina found that those with suicidal ideation, 66.0% reported persistent or recurrent ideation (60). Erika also showed that one third of young adults with a history of suicidal ideation reported suicidal ideation 4 years later (61). Zivin et al. (62) found that 35% of college students with suicidal ideation reported suicidal ideation 2 years later. Our study, along with these previous studies, indicated an important role of history of suicidal ideation in predicting future suicidal ideation. In addtion, we also found poor sleep quality was one of the most important predictors for suicidal ideation in all the three models (63–65). One possible explanation is that poor sleep quality results in being awake at night, which may cause a decrease in frontal lobe function. And decreased frontal lobe function may lead to decreased problems solving ability and increased impulsive behavior, both of which may be associated with the risk of suicide (66). Another possibility is that insomnia or nightmares may trigger perceptions of defeat, which in turn leads to feelings of entrapment, isolation and hopelessness, and ultimately suicidality (67).

Moreover, self-rated mental health has been identified to be an important predictor for suicidal ideation in our study, which was comported with prior studies (68–70). Self-reported mental health status reflects the overall mental state of college students to a certain extent. Isaac et al. (71) and Peter et al. (72) also revealed that poor self-rated mental health was a risk factor for suicidal ideation.

Apart from above, as potential predictors of suicidal ideation, our study indicated that paranoid symptom and internet addiction could not be ignored. Previous studies found that the severity of suicidal ideation was associated with higher levels of paranoia (73, 74). Paranoid people have the following characteristics that can lead to suicidal ideation: marked negative-self beliefs and low psychological wellbeing (defeat), pessimism and lack of anticipation of pleasure (entrapment), and worry (ruminative thinking) (73, 75). According to integrated motivational–volitional model of suicidal behavior, defeat and entrapment drive the emergence of suicidal ideation (75). Moreover, internet addiction is common among young students. Many studies had demonstrated that the individuals with internet addiction had significantly higher rates of suicidal ideation (76–78). Internet addiction might contribute to suicidal ideation by promoting psychiatric symptoms such as anxiety and depression through biological, psychological, or sociological mechanisms (77, 79). Besides, due to the anonymous nature of the internet, students with internet addiction have more chances to be exposed to suicidal thoughts or experiences (80, 81) and less sensitive to the adverse consequences of suicide (82, 83).

Self-rated physical health and self-rated overall health were also found by two of our models to be potential predictors of suicidal ideation. Previous studies found that physical illness especially cardiovascular disease, diabetes and cancer were more likely to result in suicidal ideation (71, 84–86). Similar results were reported by FäSSBERG, which found a person would have a high risk of suicidal ideation when the illness threatens the person's independence, sense of usefulness, sense of worth, dignity and/or enjoyment of life (87).

In the model 1, childhood emotional abuse was found to be a predictor for suicide ideation, which was consistent with previous studies (18, 88, 89). In Three-Step Model of Suicide, emotional abuse as an experience contributes to both psychological pain and hopelessness, which may lead to an elevated risk for suicide (90). The interpersonal factors such as attachment security and social-support-seeking behaviors may serve as a mediating role between childhood emotional abuse and suicide ideation (91).

In the model 2, income was identified as one of the top five predictors for suicide ideation in our study. And in the model 1, study pressure was found to be an important predictor. Some studies suggested that both high study pressure and low income could be seen as stressors for students, which might cause psychological strain leading to suicidal ideation (92, 93). However, results were not agreeable. Aqeel Khan and Marcon reported that low income was the risk factor of suicidal ideation and suicide (94, 95). But this association was not found in other studies (96–98). The same inconsistent results were observed when exploring relationship between study pressure and suicidal ideation among college students. Seo found that study pressure was a risk factor for suicide ideation (16). Wang revealed that increased study pressure and burden was associated with a higher risk of suicidal ideation (99). While in Tang et al.'s research (98), study pressure was not associated with suicidal ideation. The roles of income and study pressure in predicting suicide ideation need further study.

In conclusion, previous suicidal ideation and poor sleep quality were robust predictors for suicide ideation among college students. Other predictors were identified either in one or two of the prediction models. The three models predicted suicidal ideation of college students at different stages of their college study, respectively, indicating that there might be different risk factors for suicidal ideation at different stages. As freshmen, adaption to new environment is the main theme of their lives so that study pressure and paranoid symptom are primary issues. In the second year of college, internet addiction issue appears and self-rated mental health starts to play an important role in predicting suicidal ideation. In the third year of college self-rated overall health, self-rated mental health and self-rated physical health appear to be dominant among others. Although the underline mechanisms are not clear yet and further study is needed, our study implies that when developing strategies of suicide intervention for college students, students' stage should be taken into consideration, and students who have ever had previous suicidal ideation and poor sleep quality should be paid consistent attention to.

## Limitations

We systematically evaluated behavioral and psychological symptoms and used RBFNN for the first time to predict suicidal ideation of college students. This study adds knowledge of potential improtant behavioral and psychological symptoms that might be associated with suicidal ideation, as well as enriches the application of machine learning methods in the field of suicide research. Meanwhile, we recognize several limitations as well. First, the data were restricted to a single university with a limited number of suicidal events, potentially limiting both its power and generalizability. Second, since we did not include psychiatrists in this study to administer mental health scales, those scales couldn't be used to render a clinical diagnosis, thus limiting some of our understanding. Third, considering all the data were self-reported by respondents, it inevitably introduced reporting bias such as higher report rate of negative events among depressed respondents. Fourth, due to the rarity of a suicide event, we used suicidal ideation as proxy outcome variable. Although only a small amount of people with suicidal ideation would finally commit suicide, suicidal ideation as the first step toward suicide strongly predicted suicide. Therefore, prevention of suicidal ideation is meaningful for suicide prevention. Fifth, participants who reported previous suicidal behavior might also incline to report subsequent suicidal behavior, which might cause bias. Sixth, we only recruited those who completed all four surveys. Although there was no significant difference in the incidence of suicidal ideation between the sample population and those who were lost to follow-up, selection bias might exist.

## Conclusion

The incidence of suicidal ideation among Chinese college students was about 3.35%, which was not high comparing to the number in western countries. Our study suggested that RBFNN method was able to provide accurate prediction of suicidal ideation. Moreover, previous suicidal ideation and poor subjective sleep quality were the robust important predictors. And self-rated mental health, paranoid symptom, internet addiction, self-rated physical health, self-rated overall health, emotional abuse, income and study pressure were also identified as important predictors in one or two prediction models. We suggest that when developing strategies of suicide intervention among college students, which grade students are at should be taken into consideration, and students who have ever had previous suicidal ideation and poor sleep quality should be pay consistent attention to.

## Data availability statement

The original contributions presented in the study are included in the article/Supplementary material, further inquiries can be directed to the corresponding author/s.

## Ethics statement

The survey protocol (including the informed consent) was approved by the Medical Ethics Committee of West China Hospital of Sichuan University. All participants signed the informed consent forms.

## Author contributions

SL and YW led the analysis of the data and wrote the draft of the manuscript. XZ, QZ, XJ, and YuZ assisted with writing the draft of the manuscript. XL, WG, and YaZ participated in data collection and coordination of the study. QL contributed to the study design. TL and PQ were responsible for quality control of this study and review of the manuscript. All authors have read and approved the final version of the manuscript.

## Conflict of interest

The authors declare that the research was conducted in the absence of any commercial or financial relationships that could be construed as a potential conflict of interest.

## Publisher's note

All claims expressed in this article are solely those of the authors and do not necessarily represent those of their affiliated organizations, or those of the publisher, the editors and the reviewers. Any product that may be evaluated in this article, or claim that may be made by its manufacturer, is not guaranteed or endorsed by the publisher.
