# Supplementary material for: Prediction of suicidal ideation among Chinese college students based on radial basis function neural network
Source: Front Public Health. 2022 Dec 1;10:1042218. doi: 10.3389/fpubh.2022.1042218 (PMC9751327; doi:10.3389/fpubh.2022.1042218)
Supplement: Supplementary file 1 [file Table_1.docx]

Table S1 Comparison of the prevalence of suicidal ideation in the last twelve months between the sample population and the lost-to-follow population

| **Different stages of investigation** | ***N*** | **the prevalence of suicidal ideation (％)** | $\boldsymbol{x}^{\boldsymbol{2}}$ | ***P*** |
| --- | --- | --- | --- | --- |
| Wave 1 |  |  |  |  |
| Lost-to-follow | 15905 | 2.90 | 0.910 | 0.340 |
| Sample | 1500 | 3.33 |  |  |
| Sum | 17405 | 2.94 |  |  |
| Wave 2 |  |  |  |  |
| Lost-to-follow | 6735 | 2.84 | 0.119 | 0.731 |
| Sample | 1500 | 3.00 |  |  |
| Sum | 8235 | 2.87 |  |  |
| Wave 3 |  |  |  |  |
| Lost-to-follow | 3138 | 3.76 | 1.730 | 0.188 |
| Sample | 1500 | 3.00 |  |  |
| Sum | 4638 | 3.51 |  |  |

Figure S1. follow-up among participants
